# The Association Between HIV and Substance Use Stigma and Attitudes and Utilization of HIV Care Among Adults Who Use Injection Drugs: A Systematic Review

**DOI:** 10.1007/s10461-026-05126-1

**Published:** 2026-04-17

**Authors:** T. Dylanne Twitty, Jennifer L. Brown

**Affiliations:** 1 Department of Psychology, University of Cincinnati, Cincinnati, Ohio USA; 2 Department of Psychological Sciences, Purdue University, West Lafayette, Indiana USA

**Keywords:** HIV, Stigma, Injection drug use, Health disparities, HIV care, PrEP

## Abstract

HIV is a pressing global public health problem, with marked disparities among adults who engage in injection drug use (IDU). Despite the development and implementation of highly effective HIV prevention and treatment options, utilization and adherence are still low among those at increased risk, such as those who inject drugs. Stigma may be a significant barrier to accessing care across the HIV prevention and treatment care continuums and may be associated with poorer health outcomes. Those who engage in IDU are likely to experience stigma related to both HIV and substance use, furthering disparities among this group. A systematic literature review was conducted to examine how experiencing HIV and/or substance use stigma was associated with attitudes towards and use of HIV continuum of care outcomes and healthcare more broadly among adults who report IDU. A systematic review utilizing the Preferred Reporting Items for Systematic Reviews and Meta-Analyses (PRISMA) guidelines yielded 11 studies deemed appropriate for the current study. Experiences of stigma were examined across treatment targets (e.g., HIV prevention, ART initiation). All but two studies found that experiencing stigma was associated with poorer attitudes towards and utilization of HIV prevention and treatment. More specifically, substance use stigma was negatively associated with most HIV care continuum outcomes. A critique of the literature and directions for future research are discussed. For example, there is a need for greater consistency in stigma measurement, integrating cross-cultural and longitudinal approaches to further understand the association with the HIV care continuum among this at-risk population.

## Introduction

Human immunodeficiency virus (HIV) is a significant public health issue in countries across the globe [1]. There is increased prevalence of HIV in some parts of the world due to injection drug use (IDU), which is associated with high transmission clusters even in locations that have a historically low HIV prevalence [2–4]. Further, the high prevalence of HIV transmission and acquisition due to IDU is compounded with engagement in other risk behaviors (e.g., multiple sexual partners, transactional sex), which are common among those engaging in IDU [5, 6]. It has been estimated that those engaging in IDU have 35 times the risk of acquiring HIV than those who do not inject drugs [7]. The heightened HIV risk that IDU causes may be coupled with apprehensive and reduced utilization of HIV prevention and treatment among this population, resulting in poorer health outcomes [8–10].

The framework of HIV prevention and care continuums defines the steps to increase awareness of one’s HIV serostatus via HIV testing, HIV prevention using highly efficacious methods (e.g., pre-exposure prophylaxis; PrEP), engagement in HIV treatment, and maintenance of adherence to antiretroviral therapy (ART), resulting in viral suppression for those living with HIV [11–13]. The UNAIDS global goals are to reach their 90–90–90 target by 2030, where 90% of all people living with HIV know their HIV status, 90% of people diagnosed with HIV receive ART, and 90% of all people on HIV treatment achieve viral suppression [14]. Efforts to scale up care access have failed to adequately reach many marginalized groups, including those who inject drugs [14]. It has been found that those who engage in IDU account for almost 70% of cumulative HIV cases, but only 25% of those accessing ART [15]. While there are a multitude of contributing factors for this disparity, there is growing evidence that experiencing stigma can further disparities that are seen among those who engage in IDU [16–18]. Experiences of stigma can deter individuals, regardless of HIV status, from engaging in critical steps in the HIV care continuum (e.g., HIV testing, ART adherence), resulting in adverse health outcomes [19]. It has been estimated that those engaging in substance use have a greater risk of acquiring HIV than those who do not engage in risky substance use [7]. Not only are those who use substances at a higher risk of contracting HIV, but if they become infected, they have poorer HIV treatment outcomes should problematic substance use continue [20]. The experience of stigma, stemming from *both* one’s HIV serostatus and substance use, has been cited as an essential factor associated with adverse HIV treatment outcomes [16, 21, 22].

There have been multiple definitions and operationalizations of stigma since its conceptualization [23–25]. Stigma may encompass the internalized, interpersonal, and behavioral effects of negative views about those at risk for or living with HIV, resulting in distress and decreased healthcare engagement [26–28]. *Internalized stigma* is the degree to which a person internalizes negative beliefs or traits related to HIV or engagement in HIV risk behaviors [26]. Experiences of discrimination or prejudice because of one’s HIV status or engagement in HIV risk behaviors are defined as *enacted stigma* [26]. *Anticipated stigma* is the expectation of discrimination or prejudice based on what you have heard of or seen from others’ experiences [26]. Stigma can be HIV-specific and directed at those who are living with HIV [26]. A person can internalize negative beliefs about themselves, or others can give negative labels (e.g., guilty, disgusting, shameful) due to their assumed violation of social mores, such as engagement in behaviors that are known to increase the likelihood of HIV infection [26]. One such commonly stigmatized HIV risk behavior is engaging in substance use [29]. Substance use stigma is widely cited as a distinct manifestation of stigma with characteristics that vary based on the substance used and the community [29].

Within HIV care and especially for those who endorse lifetime substance use, engagement in associated HIV risk behaviors (e.g., IDU) can cause devaluation of oneself or from others, resulting in perceived negative judgments from others [26]. For example, having been denied healthcare services in the past (i.e., enacted stigma) may increase the likelihood of avoiding HIV testing among those who inject drugs [30]. Those with a history of or current IDU often anticipate experiencing stigma from healthcare providers, take steps to avoid it (e.g., delaying care, not disclosing one’s HIV serostatus), putting them at an increased risk of adverse outcomes [31]. It is also known that engaging in substance use is a barrier to reaching optimal viral suppression, and SU stigma being a barrier to SU treatment creates a cyclical pattern of poor care utilization [21, 32]. Since individuals at-risk for HIV or living with HIV who inject drugs may experience both HIV- and substance use-related stigma, this review examined the association between both forms of stigma and included the PrEP care continuum outcomes among individuals with current or past IDU.

### Rationale for Current Study

To date, there have been no reviews synthesizing the association between experiencing stigma and HIV prevention and treatment continuum outcomes among adults who endorse lifetime or current IDU. Considering what is known about the significant risk for HIV acquisition and transmission and stigmatizing experiences that those who have injected drugs face, it is critical to better understand these differential experiences and their potential association with HIV testing, initiation, and adherence to PrEP or ART. The current study will further investigate the role of HIV stigma and SU stigma on HIV care outcomes among those who inject drugs, as an at-risk group who may experience both stigmas and engage in behaviors that carry a high risk of HIV contraction and contribute to low utilization of HIV care. The current study examines whether specific types of stigma (i.e., HIV or substance use) are associated with: (a) attitudes towards HIV testing, PrEP use, and ART use, (b) initiation and adherence to PrEP, (c) initiation and adherence to ART, and (d) broader healthcare experiences among adults who report a history or current engagement in IDU. A methodological critique of the literature and future directions for examining the association between stigma and HIV care continuum outcomes among individuals who inject drugs will also be discussed.

## Methods

### Search Strategy and Study Selection

**Fig. 1:**
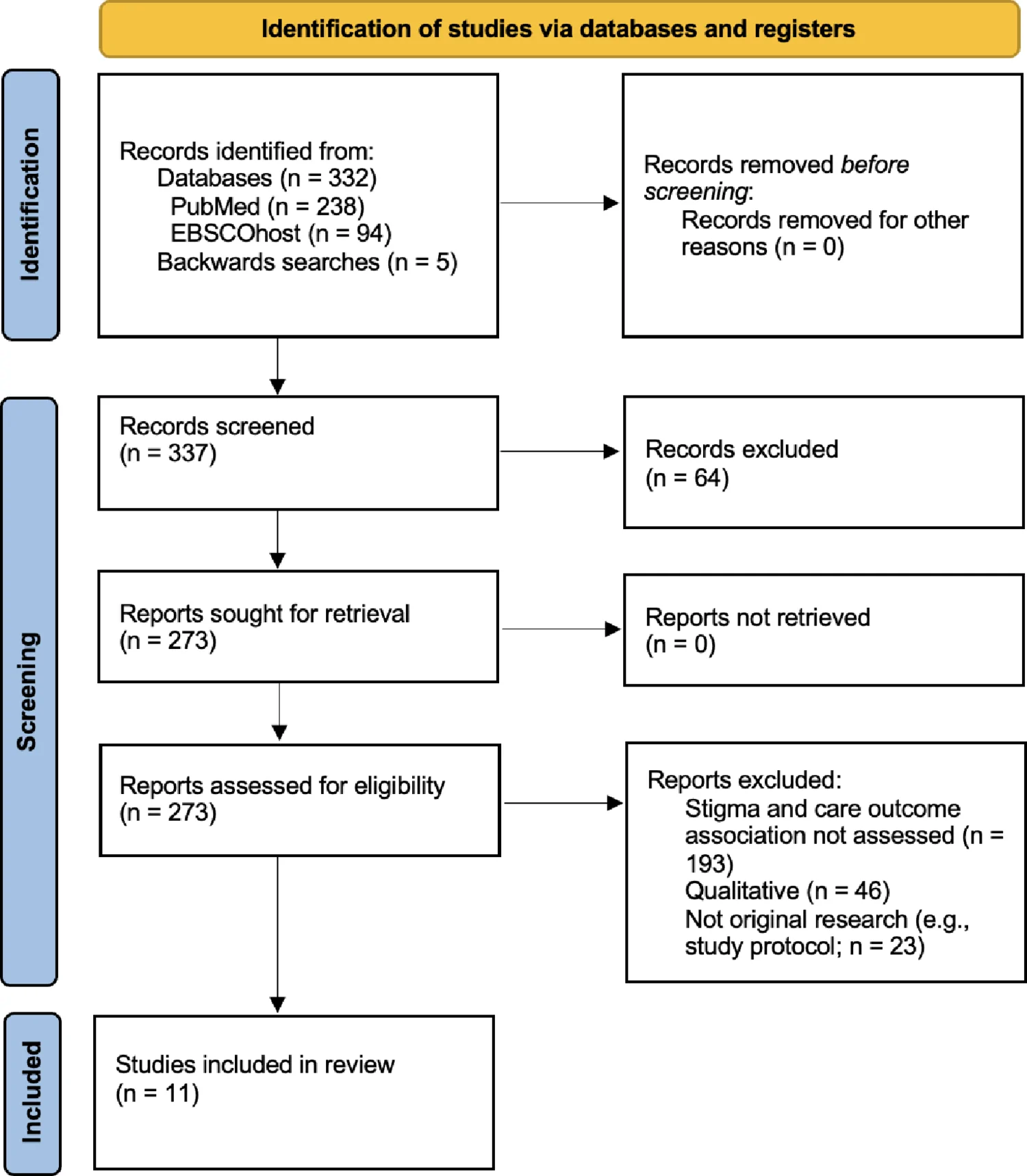
PRISMA flowchart diagram

This study utilized a standard systematic review procedure following the Preferred Reporting Items for Systematic Reviews and Meta-Analyses (PRISMA) guidelines [33]. Databases were searched to assess whether experiences of stigma were associated with attitudes towards or utilization of HIV care continuum outcomes (e.g., PrEP initiation and ART adherence) or healthcare more broadly among adults with current or past IDU. One researcher searched two electronic databases (i.e., PubMed and EBSCOhost) for published peer-reviewed literature. All searches were conducted between September 17th and November 24th, 2024, with date of publication not used as an eligibility criterion. Each database was searched with keywords oriented to find studies that examined whether stigma from HIV and/or substance use was associated with utilization or attitudes towards care across the HIV care continuums (i.e., prevention and treatment) among adults who report a history or current engagement in IDU. Briefly, the core search terms included constructs of stigma (e.g., social stigma, stigma) AND attitudes towards OR access OR adherence to care (e.g., HIV testing, ART) AND IDU (e.g., intravenous drug use, injection drug abuse). Additional articles were extracted from the references of included articles and relevant reviews. See Fig. 1 for the PRISMA diagram detailing the literature screening process.

### Inclusion and Exclusion Criteria and Study Selection

Included studies were English, full-text, peer-reviewed articles that reported on findings on the association between experiences of HIV or SU stigma and attitudes and engagement in HIV-associated care continuum outcomes (i.e., HIV testing, PrEP, ART) or healthcare more broadly among adults who report a history of or current IDU. Studies were included if they assessed experiences of HIV and/or SU stigma quantitatively among adults who were at risk of or living with HIV. To be eligible, the study had to assess whether experiencing stigma was associated with perceptions of or engagement with HIV care. Studies were excluded if they: (a) only included adolescents, (b) were dissertations, books, study protocols, and other sources without published results, or (c) did not assess stigma quantitatively.

### Reference Screening and Data Synthesis

A total of 332 results were identified and subsequently screened by title and abstract for eligibility. Backward searches were also conducted after the initial search was completed (e.g., search references from included articles and relevant reviews). Papers that met initial eligibility criteria by title and abstract were then retrieved and assessed for eligibility. After screening and review, 11 studies were included. Relevant data were extracted from each of the included studies, including study characteristics (e.g., participant characteristics, study methodology), stigma assessment (e.g., stigma measure used), HIV care continuum or healthcare outcome(s), and study findings (e.g., the association between stigma and HIV treatment/healthcare engagement).

## Results

### Study Characteristics and Methodologies

Table 1 summarizes the studies’ locations, methodologies, recruitment settings (e.g., community-based), participant inclusion criteria, sample sizes, and participant characteristics. Eight of the studies were conducted outside of the United States [8, 9, 16, 17, 30, 34–37]. The majority were based in Eastern European countries (i.e., Russia, Estonia, Ukraine) [9, 16, 35, 37]. The two studies based in the United States were conducted in the West and the South [10, 18]. One study compared differences between countries [16]. All studies utilized a cross-sectional design to examine the association between stigma and HIV care continuum outcomes. Even though the primary study conducted by Vetrova and colleagues [37] was longitudinal, substance use stigma and ART initiation were only assessed at one time point.

### Operationalization of Stigma and HIV Prevention, HIV Treatment, and Healthcare Outcomes

Table 2 overviews the HIV care continuum or healthcare outcome(s), stigma type(s), stigma measure(s), and a summary of relevant findings about the association between stigma and HIV care continuum or healthcare outcomes. The studies reviewed focused on different outcomes along the HIV care continuum (e.g., HIV testing, PrEP initiation, ART adherence). Five of the studies included HIV testing or HIV prevention methods as outcomes [10, 17, 18, 30, 34]. Two studies assessed PrEP attitudes (i.e., willingness, interest) as an outcome [10, 34]. Two studies assessed HIV testing as an outcome [17, 30]. One study assessed PrEP initiation [18]. No studies assessed PrEP adherence. Three studies assessed HIV care adherence [9, 16, 35]. Two studies assessed ART initiation or recent use [36, 37]. One study assessed general healthcare access and adherence as outcomes [8].

Two studies assessed one form of stigma (i.e., enacted stigma) [8, 30]. Three studies assessed HIV and substance use stigma individually, asking questions to measure HIV and substance use stigma distinctly [9, 16, 18]. Four studies focused exclusively on substance use stigma [8, 18, 34, 36]. Further, one of these studies focused on stigma specific to those who engage in IDU [34]. Only one study focused solely on HIV stigma [17]. Additionally, one study assessed intersectional stigma via a composite of HIV and substance use stigma [35]. Three studies utilized a psychometrically validated measure to assess HIV or substance use stigma using the original, validated form [9, 16, 37]. Most studies adapted stigma measures previously used to better meet their study objectives [9, 10, 17, 18, 34, 36, 37]. For example, Calabrese and colleagues [9] adapted the *Internalized AIDS-Related Stigma Scale* questions to also assess internalized drug stigma (e.g., “It is difficult to tell people that *I am a drug user*”). Two studies developed their own measures to assess HIV or substance use stigma [8, 30]. Further details about the stigma measures are discussed in Table 2.

### Stigma’s Association with HIV Care Continuum and Healthcare Attitudes and Utilization

Broadly, across studies, experiencing greater levels of HIV and/or substance use stigma was negatively associated with attitudes towards or utilization of HIV prevention, HIV treatment, or more general healthcare access or adherence. Nine of the 11 studies found that HIV or substance use stigma was correlated with poorer attitudes to HIV prevention or treatment (e.g., interest in initiating PrEP) or HIV treatment engagement (e.g., ART initiation) or other healthcare use [8–10, 16–18, 30, 35–37]. A minority of studies found that HIV or substance use stigma was not associated with attitudes towards (i.e., PrEP willingness) or use of healthcare [16, 31].

Several studies examined subtypes of HIV and substance use stigma (e.g., intersectional stigma, enacted stigma) in relation to HIV care continuum and healthcare outcomes (see Table 2) [16–18, 34–37]. However, some studies collapsed the subscales and focused on the broader construct of HIV or substance use stigma in analyses [34, 37]. For studies that did not collapse these subscales in their analyses, findings varied across stigma mechanisms. Five studies found that enacted stigma (e.g., enacted substance use stigma) was associated with lower levels of HIV testing, lower interest in PrEP, reduced odds of ART use, decreased HIV treatment adherence, and non-adherence to healthcare in the past year [8, 10, 30, 35, 36]. Three studies found that internalized stigma of HIV and drug use was negatively associated with HIV treatment adherence [9, 16, 35]. One study found that anticipated HIV stigma was negatively associated with ART adherence [16]. Two studies found that intersectional stigma (e.g., a composite of HIV and substance use stigma) was associated with poorer access to care (i.e., affordability, availability, convenience, and specialist availability) and lower odds of HIV treatment adherence [35, 37]. These findings suggest a potential combined effect of *both* HIV and SU stigma on HIV care engagement and healthcare more broadly.

The methodologies and data analytic approaches employed to examine the relationship between stigma and HIV care varied across studies. Two studies assessed whether experiencing stigma deterred care use (i.e., PrEP initiation, any healthcare) with dichotomous substance use stigma items [8, 18]. For example, Suprasert and colleagues [18] found that 56% of participants reported delaying or avoiding healthcare services due to experiencing any IDU stigma in the past year. The remaining studies utilized regression analyses to examine the association between stigma and the care outcome [9, 10, 16, 17, 30, 35–37]. The majority used logistic regression analyses with a binary care outcome (e.g., 0: non-adherence to ART, 1: adherence to ART) [17, 30, 35, 36]. One study examined whether the association between internalized and anticipated HIV and substance use stigma and HIV treatment adherence differed between participants in Russia and Estonia [16]. Burke and colleagues found that internalized HIV and substance use stigma and anticipated HIV stigma were negatively associated with HIV treatment adherence in Russia, but not in Estonia [16]. Overall, findings consistently showed that experiencing greater overall stigma and associated stigma mechanisms were negatively associated with attitudes towards or use of HIV care and healthcare more broadly.

## Discussion

This systematic review examined the associations between experiencing higher levels of stigma and attitudes towards or engagement (i.e., use or adherence) in HIV testing, PrEP, ART, or general healthcare among adults endorsing IDU. The reviewed studies further our knowledge about how different forms of stigma may be related to HIV care utilization across the care continuums and healthcare more broadly. Across studies, this literature suggests that experiencing heightened stigma is associated with poorer attitudes towards or engagement in HIV prevention and treatment or healthcare more broadly. It was found that enacted stigma, enacted substance use stigma in particular, was most consistently associated with poorer outcomes across the continuum of HIV care [8, 10, 30, 36]. One contextual example to illustrate has been that those who are living with HIV may use substances to cope with stress related to HIV, which further harms their ability to adhere to treatment [38].

With regards to prevention, studies examined the relation between stigma and PrEP attitudes and initiation, but no studies included PrEP adherence as an outcome. While PrEP access is often limited in many parts of the world [36], PrEP options have continued to expand [39]. Thus, it will be important to understand how stigma could be associated with PrEP attitudes, initiation, and adherence. Even though those who are engaging in HIV preventative services may not experience stigma associated with living with HIV, they may be affected by stigma linked to their substance use or IDU. Across studies, substance use stigma was associated with decreased HIV care engagement [8–10, 16–18, 30, 35–37]. Substance use treatment has been historically under-resourced, with those who struggle with addiction facing reduced access to healthcare resources and legal protections compared to those who struggle with other mental health challenges [40]. In addition, individuals who use drugs experience stigma due to IDU in addition to HIV stigma [27]. These findings highlight the marginalization that individuals who use substances face regardless of their HIV status, which can impact their access to critical HIV testing, prevention, and treatment.

While the breadth of care outcomes included HIV treatment as a target, there are still gaps in our understanding of stigma’s association with HIV treatment initiation and adherence among this population. While HIV care adherence was the most common care type examined as an outcome across studies [9, 16, 35], initiation of HIV treatment was minimally represented among the studies [37]. Initiation of ART is a critical first step in HIV treatment, but has been found to be low among those who inject drugs [41, 42]. Due to recent HIV outbreaks among communities of those engaging in IDU, eliminating barriers to improve uptake and adherence among this at-risk group is an important future direction [43]. Experiencing substance use stigma can cause a delay in addiction care seeking, specifically or any healthcare altogether [44, 45]. For those living with HIV, HIV stigma may cause individuals to delay ART initiation [46, 47]. Both circumstances may result in individuals experiencing more severe adverse outcomes and the spread of HIV throughout a community [47, 48].

Most studies were conducted across diverse geographic locations, underscoring the importance of cultural context in interpreting findings. The majority of studies were conducted in European countries, with Russia being the most common country site. Despite the high prevalence of HIV in Sub-Saharan Africa, there were no studies conducted examining stigma and HIV care outcomes among those with a history of IDU [49]. For example, the Institute for Health Metrics and Evaluation (IHME) found that HIV accounted for over 30% of the burden of disease in South Africa in 2015 [50, 51]. Thus, there is a need to examine stigma in relation to HIV care continuum outcomes in locales with the highest HIV disease burden. In addition, the conceptualization of stigma may be culturally bound, necessitating the cultural adaptation of measures for their study populations (e.g., language, substance use characteristics, operationalization of care adherence) [16, 34–36, 52, 53]. It is recommended that additional efforts be directed to understanding stigma’s influence on HIV care continuum use in these understudied contexts using measures that capture their unique cultural context of HIV and substance use stigma [54, 55].

There were differences in how stigma was conceptualized, the terms used to describe forms of stigma, the approach to measuring stigma, and the extent to which measures were culturally adapted (e.g., language and substance use behaviors). Thus, there was substantial heterogeneity in how stigma was measured across studies. An added limitation of the studies was the low rigor of the stigma assessment tools utilized. Among studies that developed their own measures without a systematic psychometric process, one cited preliminary research as the foundation for the questions [34]. Across studies, when researchers used measures with subtests (e.g., internalized stigma, HIV-specific stigma) or incorporated multiple measures, they were better able to capture finer-grained levels of stigma mechanisms [10, 16, 18, 35]. Enacted stigma was the most common stigma mechanism studied and was found to be associated with reduced use of care across the HIV prevention and treatment continuums (e.g., HIV testing avoidance, non-adherence to HIV care) as well as healthcare more broadly (e.g., healthcare access) [8, 10, 30, 35]. While many studies targeted different mechanisms of stigma (e.g., anticipated, internalized, and community), other types of stigma, such as additive or compounding, were not explicitly studied.

Additionally, the two studies that examined what was labeled ‘intersectional’ stigma used this term to define a composite variable of HIV and SU stigma [35, 37]. This operationalization of stigma from the studies found in the review differs from how the term *intersectional stigma* is often characterized more broadly to include the concept that one’s life is impacted by the converging of stigma based on social identity (e.g., race, gender, class), practices (e.g., drug use) and health statuses (e.g., HIV serostatus) [56]. However, the concept that one can hold multiple stigmatized identities and associated structural inequities is overlapping and distinct in HIV stigma and SU stigma [35, 57]. Even with a more isolated approach, differences in operationalization still affect our ability to interpret and compare findings.

Future research should consider approaches to move towards more consistent operational definitions and measurement of stigma mechanisms. Further, there is a need for studies to examine the psychometrics of HIV and substance use stigma measures, including those that have been culturally adapted. For example, in the NIH-recommended measures listed in the *PhenX Toolkit* [58], the only available English translations for all protocols/questionnaires were in Chinese and Spanish. It is recommended that researchers use psychometrically validated measures and common databases, such as the *PhenX* database, for their scientific rigor. Many studies utilized adapted versions of measures to align with the cultural context of their population [9, 10, 35]. When developing or adapting a measure, it is recommended that researchers incorporate theoretical frameworks (e.g., the *HIV Stigma Framework*) and subscales to capture the mechanisms of stigma [27, 40].

An additional methodological limitation across studies was the use of a cross-sectional, correlational design. The use of cross-sectional studies limits our ability to assess the temporal nature of stigma experiences and attitudes towards, use of, and adherence to care [8]. It is recommended that study designs are longitudinally designed to assess whether participants who perceive experiencing HIV or substance use stigma initiate or adhere to a form of care at follow-up(s) to establish the temporal relationship between stigma and HIV care outcomes and how variability in stigma experiences over time is associated with HIV care cascade outcomes. This would be important to understand in order to inform interventions that aim to improve use and adherence to HIV testing, prevention, and treatment [59].

## Conclusions

HIV disparities are heightened among marginalized populations, including those engaging in IDU. Due to the intersecting stigmas related to HIV and substance use, those who engage in IDU face additional barriers to HIV care and healthcare more broadly. The current study examined the association between experiencing different types of stigma (i.e., HIV and substance use) and attitudes towards and use of HIV testing, PrEP, and ART among adults endorsing a history of or current IDU. Overall, most studies found that experiencing heightened stigma was associated with poorer attitudes towards or use of HIV testing or care and healthcare more broadly. This association was most often seen involving substance use stigma. However, due to the heterogeneous nature of the studies (e.g., operationalization of stigma, validation of stigma measures, and care outcome types), it is important for future work to address gaps that would enable standardized comparisons across settings and populations. Key recommendations to advance this literature include directing efforts towards study settings in underserved countries that are not well-represented in this literature, establishing a validated, comprehensive measure to assess both HIV and substance use stigma cross-culturally, and targeting PrEP adherence as a care outcome. This review highlights gaps for future work to gain knowledge about the relation between stigma and HIV care continuum outcomes to enhance HIV prevention and treatment services among those who engage in injection drug use.

**Table 1:** Study characteristics

Author/year	Location	Recruitment Setting	Inclusion criteria	*N*	Sample characteristics
Belludi et al. [34]	India	Community-based (12 different cities as sites)	Spoke English, Hindi or one of the 7 regional languages Report IDU in the past 2 years	10,538	Ages: 25–35 years (median 28 years) Never married: 48.3% Completed up to secondary education: 69.3%
Brener et al. [8]	Australia	Community-based (IDU organizations) (secondary data analysis)	Women Endorsing IDU	232	Ages: 18–65 years (mean 41.84 years) Completed up to secondary education: 11.7% Unemployed: 58.9%
Burke et al. [16]	Russia or Estonia	Community-based	Reported IDU in the past 4 weeks Spoke Russian or Estonian	Russia: 381 Estonia: 288	All participants knew they were HIV positive *Russia*: Mean age: 32.92 years Male: 79% Up to secondary education: 32% Facing financial difficulties: 77% *Estonia*: Mean age: 30.58 years Male: 73% Up to secondary education: 20% Facing financial difficulties: 84%
Calabrese et al. [9]	St. Petersburg, Russia	Community-based (secondary data analysis)	IDU in the past 4 weeks Living with HIV Residing in St. Petersburg	383	Age: 74.1% aged 30 years and up Male: 79.1% Up to a secondary education (through 11th grade): 31.6% Facing financial difficulty: 77.3%
Noroozi et al. [17]	Tehran, Iran	Community-based	Injected drugs once per month for the last 6 months Resided in Tehran, Iran	500	Ages: 19–67 (median 32 years) At least secondary school (6 or more years): 27% Unemployed: 78% Monthly income below 150 USD: 76%
Owczarzak et al. [35]	Ukraine	Community-based (outreach, service centers)	Female Living in Kyiv City Past 3-month IDU HIV positive (confirmed with a rapid test)	306	Age: median of 34 years Female: 100% Did not have enough money to meet most financial needs: 91% Regular employment: 36% Vocational or higher education: 64%
Patel et al. [36]	India	Community-based (secondary data analysis)	Self-reported IDU in past 2 years HIV positive (confirmed with a rapid test) Eligible for ART	971	Age: median age 36 years Male: 76.8% High school or more: 20.7%
Suprasert et al. [60]	United States	Community-based	English or Spanish Past-year IDU Residing in San Francisco or San Mateo, counties in California	527	Age: 46.2% aged 50 years or more 67.2% male High school or general education degree: 41.9% Unemployed: 60.5%
Surratt et al. [10]	United States	Community-based	Minimum of one occasion of past-month IDU Past 6-month PrEP initiation Residing in Clay or Cox County in Kentucky	80	Age: mean 39.7 years Female: 52.5%
Ti et al. [61]	Thailand	Community-based	Residing in Bangkok or adjacent provinces HIV negative or unknown serostatus	440	Age: median of 38 years (IQR: 34–48) Female: 19.4%
Vetrova et al. [37]	Russia	Not specified (secondary data analysis)	18–70 years Documented HIV infection No ART initiation by baseline Ability to provide two contacts to aid with follow-up Stable address within 100 km of St. Petersburg Possession of phone Completed HIV and substance use questionnaire	188	Age: mean of 33.5 years Male: 67.6% Secondary school education: 76.6% Employed: 46.8%

*AIDS* Acquired Immunodeficiency Syndrome, *HIV* Human Immunodeficiency Virus, *IDU* Injection Drug Use, *PrEP* Preexposure Prophylaxis, *ART* antiretroviral therapy

**Table 2:** Healthcare and stigma characteristics and findings

Author/year	HIV care continuum or healthcare outcome(s)	Stigma type(s)	Stigma measure(s)	Relevant findings for the association between stigma and HIV care continuum or healthcare outcomes
Belludi et al. [34]	PrEP willingness	Injection drug use stigma with sub-scales (experienced, vicarious, community, self-stigma)	Measure adapted by Stewart et al. [62]	In a descriptive analysis, 19.9% endorsed concerns that “people may think I have HIV/AIDS” as a reason of being unwilling to use oral PrEP. In univariable multi-level logistic regression analyses, unadjusted odds ratio OR = 1.02 (0.95–1.09), *p* > .05, found that stigma (composite score calculated) was not associated with willingness to use oral PrEP.
Brener et al. [8]	Any healthcare access or adherence	Enacted injection drug use stigma	Self-report (2 items): 1. past 12-month experience of stigma or discrimination related to their IDU 2. report of negative or different treatment by health workers (due to their IV drug use)	In descriptive analyses, among women who inject drugs, 27.3% endorsed being treated negatively by healthcare workers often or always in the past year. A multiple, unadjusted linear regression model found that patient sample reported that past 12-month negative treatment by a healthcare worker due to IDU significantly predicted reduced access to healthcare (F = 5.190, *p* < .0001, R^2^ = 0.332).
Burke et al. [16]	ART adherence	Internalized and anticipated HIV stigma Internalized and anticipated drug stigma	*Internalized AIDS-Related Stigma Scale* [63] *Stigma Consciousness Questionnaire* [64]	Russia: Descriptive analyses found that, of adults using injection drugs, 68% reported not receiving regular care. Bivariate correlational analyses found that internalized drug use stigma (B = -0.35, *p* < .01), internalized HIV stigma (B = -0.43, *p* < .01) and anticipated HIV stigma were negatively associated with HIV care adherence (B = -0.15, *p* < .01). Estonia: Descriptive analyses found that, of adults using injection drugs, 30% reported not receiving regular care. Bivariate correlational analyses did not find that internalized drug stigma (B = 0.03, *p* > .05), internalized HIV stigma (B = -0.06, *p* > .05), anticipated drug stigma (B = 0.07, *p* > .05) or anticipated HIV stigma (B = -0.04, *p* > .05) were not associated with HIV care adherence.
Calabrese et al. [9]	HIV-related care adherence	Internalized HIV stigma and internalized drug stigma	*Internalized AIDS-Related Stigma Scale *[63] Internalized substance use stigma assessed with adapted *Internalized AIDS-Related Stigma Scale* [63]	Descriptive analyses found that, of people living with HIV who inject drugs, 32.4% adhere to HIV care and 11.2% adhere to drug treatment. Simple slopes analyses, adjusting for sociodemographic variables and social/behavioral history, found a negative association between internalized HIV stigma and likelihood of receiving regular HIV care (1 + visits/6 months) among those who also experienced high (not low) internalized drug stigma (*b* = -1.62, *SE* = 0.30, *AOR* = 0.20 [0.11, 0.36], *p* < .001).
Noroozi er al. [17]	HIV testing	HIV stigma (shame/blame/isolation, perceived discrimination and equity subscales)	Measure previously developed by Genberg et al. [65]	Multivariate logistic regression analyses found that, of those using injection drugs, lower levels of HIV-related stigma increased almost 3 times the lifetime odds of HIV testing (*OR* = 2.78, 95% *CI* 1.15–5.2).
Owczarak et al. [35]	HIV care adherence (two HIV visits more than 3 months apart within the last year or if they had one HIV visit within 3-months of their study visit - *Health Resources and Services Administration* definition of care retention)	Internalized drug use stigma Enacted drug use stigma Internalized HIV stigma Enacted HIV stigma Internalized intersectional HIV and drug use stigma Enacted intersectional drug use and HIV stigma	Measure adapted from preliminary science [66]	Using univariable logistic analyses, among women living with HIV using injection drugs, those reporting internalized drug use stigma had 56% lower odds of care engagement than those who reported no internalized drug use stigma (*OR* = 0.44, 95% *CI* = 0.21–0.91, *p* < .05). Using univariable logistic analyses, those reporting internalized HIV stigma had 43% lower odds of care engagement than those who reported no internalized HIV stigma (*OR* = 0.57, 95% *CI* = 0.24–1.36, *p* > .05). Using univariable logistic analyses, among this sample, those reporting internalized intersectional stigma had 62% lower odds of care engagement compared to those who reported none (*OR* = 0.38, 95% *CI* = 0.22–0.65, *p* < .001). Using univariable logistic analyses, among this sample, those reporting enacted intersectional stigma had 49% lower odds of care engagement compared to those who reported none (*OR* = 0.51, 95% *CI* = 0.27–0.96, *p* < .05).
Patel et al. [36]	ART use (past 30 days)	Enacted substance use stigma Internalized substance use stigma	Measure developed by Patel et al. [67]	Univariate logistic regression analyses found that among those using injection drugs, those reporting enacted stigma (versus none) had 1/4 adjusted odds of ART use and 1/2 lower adjusted odds of viral suppression (*OR* = 0.47, 95% CI = 0.31–0.71). Univariate logistic regression analyses found that reporting a high degree of internalized stigma was associated with 61% lower crude odds of viral suppression (*OR* = 0.61, 95% CI = 0.37–0.996). Univariate logistic regression analyses found that experiencing any enacted stigma was associated with lower odds of viral suppression among those at least 35 years old but not less than (*OR* = 0.28, 95% CI = 0.14–0.58). Similarly, univariate logistic regression analyses found that high levels of internalized stigma were associated with lower adjusted odds of viral suppression (*OR* = 0.44, 95% CI = 0.27–0.73).
Suprasert et al. [18]	PrEP initiation	Enacted injection drug use stigma Perceived/anticipated injection drug use stigma	*National HIV Behavioral Surveillance* measure extracted from [68]	Descriptive analyses found that, among those with a history of IDU, 56% reported delaying or avoiding health care or other services due to perceived injection drug use stigma in the past 12 months.
Surratt et al. [10]	PrEP interest	Community HIV stigma Enacted substance use stigma	Community HIV stigma: abbreviated form of *Berger HIV Stigma Scal*e [71] Enacted substance use stigma: *dignity denial* item adapted by [70]	In analyses of bivariate predictors, community HIV stigma was statistical different in participants endorsing low and high PrEP interest (*p* = .02). In analyses of bivariate predictors, past year enacted substance use stigma was also statistically different between low and high PrEP interest. Logistic regression model analyses found that, among those endorsing recent IDU, enacted substance use stigma was significant in unadjusted and adjusted regression models. Unadjusted logistic regression analyses found that community HIV stigma and enacted substance use stigma were associated with PrEP interest.
Ti et al. [30]	HIV testing	Enacted substance use stigma	Single item developed for this study (i.e., “Do you sometimes avoid accessing HIV tests because you are a drug user?”)	Using logistic regression analyses, among a community sample, having been declined healthcare services increased the likelihood of avoiding HIV testing (*OR* = 6.72, 95% CI 3.06–14.74).
Vetrova et al. [37]	ART initiation	HIV stigma with sub-scales (internalized stigma, anticipated stigma, enacted stigma) substance use stigma with subscales (internalized stigma, anticipated stigma, enacted stigma) intersectional stigma	Abbreviated *Berger HIV Stigma Scale* [71] *Substance Abuse Self-Stigma Scale* [72] intersectional stigma: 4-category composite variable HIV and substance use stigma	Using logistic regression analyses, among those living with HIV, high HIV stigma was not associated with poor access to past year healthcare services in unadjusted or adjusted models (*OR* = 0.71 95% CI 0.41–2.10, *p* > .05; *aOR* = 1.08; 95% CI 0.70–1.66, *p* > .05). Additional logistic regression analyses showed that high HIV stigma was not associated with access to HIV care in the unadjusted or adjusted analyses (*OR* = 0.71, 95% CI 0.36–1.40, *p* > .05; *aOR* = 0.73; 95% CI 0.45–1.19, *p* > .05). Using logistic regression analyses, among those living with HIV, high SU stigma was associated with poor access to past year healthcare services in unadjusted and adjusted models (*OR* = 1.85, 95% CI 1.02–3.39, *p* < .05; *aOR* = 2.31; 95% CI 1.50–3.57, *p* < .0001). Additional logistic regression analyses, showed that high SU stigma was not associated with access to HIV care in the unadjusted or adjusted analyses (*OR* = 1.08, 95% CI 0.55–2.10, *p* > .05; aOR = 0.86; 95% CI 0.54–1.35, *p* > .05). Adjusted logistic regression analyses, among those living HIV, high intersectional stigma was associated with poor access to healthcare in the past year (*OR* =1.78, 95% CI 0.82–3.90, *p* > .05; *aOR* = 1.86; 95% CI 1.192–2.92, *p* < .01. Additional logistic regression analyses showed that high intersectional stigma was not associated with past 3-month HIV care access (*OR* = 0.84, 95% CI 0.36–1.98, *p* > .05; *aOR* = 0.92; 95% CI 0.56–1.49, *p* > .05).

*AIDS* Acquired Immunodeficiency Syndrome, *HIV* Human Immunodeficiency Virus, *IDU* Injection Drug Use, *PrEP* Preexposure Prophylaxis, *ART* antiretroviral therapy
